# Conditioning Increases the Gain of Contraction-Induced Sarcolemmal Substrate Transport in Ultra-Endurance Racing Sled Dogs

**DOI:** 10.1371/journal.pone.0103087

**Published:** 2014-07-30

**Authors:** Michael S. Davis, Arend Bonen, Laelie A. Snook, Swati S. Jain, Kenneth Bartels, Raymond Geor, Karsten Hueffer

**Affiliations:** 1 Department of Physiological Sciences, Oklahoma State University, Stillwater, Oklahoma, United States of America; 2 Department of Human Health and Nutritional Sciences, University of Guelph, Guelph, Ontario, Canada; 3 Department of Veterinary Clinical Sciences, Oklahoma State University, Stillwater, Oklahoma, United States of America; 4 Department of Large Animal Clinical Science, Michigan State University, East Lansing, Michigan, United States of America; 5 Department of Veterinary Medicine, University of Alaska-Fairbanks, Fairbanks, Alaska, United States of America; Rosalind Franklin University, United States of America

## Abstract

Endurance exercise relies on transsarcolemmal flux of substrates in order to avoid depletion of intramuscular reserves. Previous studies of endurance trained sled dogs have shown a remarkable capacity of these dogs to adapt rapidly to endurance exercise by decreasing the utilization of intramuscular reserves. The current study tested the hypothesis that the dogs' glycogen-sparing phenotype is due to increased sarcolemmal transport of glucose and fatty acids. Basal and exercise-induced transport of glucose and fatty acids into sarcolemmal vesicles was evaluated in racing sled dogs prior to and after 7 months of exercise conditioning. Sarcolemmal substrate transport capacity was measured using sarcolemmal vesicles and radiolabelled substrates, and transporter abundance was measured using Western blot quantification in whole muscle homogenates and the sarcolemmal vesicle preparations. Conditioning resulted in increased basal and exercise-induced transport of both glucose and palmitate. Neither acute exercise nor conditioning resulted in changes in muscle content of GLUT4 or FAT/CD36, but conditioning did result in decreased abundance of both transporters in the sarcolemmal vesicles used for the basal transport assays, and this decrease was further amplified in the vesicles used for the exercise-induced transport assays. These results demonstrate conditioning-induced increases in sarcolemmal transport of oxidizable substrates, as well as increased gain of exercise-induced sarcolemmal transport of these substrates. These results further indicate that increased sarcolemmal transport of oxidizable substrates may be due to either an increased intrinsic capacity of the existing transporters or to a different population of transporters from those investigated.

## Introduction

Metabolizable substrate uptake in skeletal muscle is regulated in order to meet the dual roles of the tissue of energy storage during periods of overall positive energy balance and energy expenditure during muscular work. Diffusion of the key metabolizable substrates across the sarcolemma is either limited (fatty acids) or virtually non-existent (glucose), and therefore sarcolemmal expression of substrate-specific transporters provides a key point of regulation [1], [2]. Dynamic expression of transporters such as GLUT4 and fatty acid translocase (FAT)/CD36, controlled by the appropriate intracellular and extracellular signals, provides a mechanism for a large range of transsarcolemmal flux that is responsive to acute changes in the need for metabolizable substrate uptake. Both GLUT4 and FAT/CD36 are stored intracellularly, but will translocate to the sarcolemma during periods of contraction or insulin stimulation. In both cases, increased transsarcolemmal flux of their respective substrates occurs with increased sarcolemmal localization of these transporters.

Endurance-trained racing sled dogs have been shown to sustain large caloric expenditures during competition (nearly 12,000 kcal/day or over 4000 kJ/kg^0.75^/day) [3], indicating a tremendous capacity to transport oxidizable substrates into working muscle cells. However, this capacity appears to be highly dynamic and capable of rapid increases within the context of a single multiday exercise challenge. In previous studies in which trained racing sled dogs performed daily controlled exercise sessions, the dogs initially depleted intramuscular glycogen and triglyceride, but transitioned from glycogen-depleting to glycogen-sparing over 96 hrs of continued intermittent exercise [4], [5]. With the overall workload (speed, distance, duration, load) held constant, we hypothesized that the overall energetic requirements of the exercise were similarly constant and that the sparing of intramuscular glycogen was made possible by increased availability of blood-borne oxidative substrates. Therefore, we conducted a set of experiments to test the hypothesis that the development of a glycogen-sparing, fatigue-resistant phenotype is the result of increased sensitivity of exercise-induced translocation of substrate transporters to the sarcolemma.

## Materials and Methods

This study was carried out in strict accordance with the recommendations in the Guide for the Care and Use of Laboratory Animals of the National Institutes of Health. The protocol was approved by the Institutional Animal Care and Use Committee of Oklahoma State University (Permit Number: 27-2956). All surgery was performed as described under general anesthesia, post-operative pain management was provided to all subjects, and all efforts were made to minimize suffering.

Thirty-seven mature sled dogs were used in the study, and assigned randomly into one of 5 groups. Six dogs were examined as unconditioned sedentary controls, and had not had any compulsory exercise for at least 4 months. Eight additional unconditioned dogs were examined immediately following a treadmill standardized exercise test (25 min at 6.5 mph and 4.5% incline). Eight dogs were examined as conditioned sedentary controls and had completed a full season (7 months) of endurance conditioning and racing, including a single 1000 mile race completed approximately 2 weeks prior to the examination, but had not undergone any compulsory exercise for at least 96 h prior to examination. Eight dogs with the same conditioning, racing, and recent exercise history were examined immediately following the same treadmill standardized exercise test as used for the unconditioned dogs. Seven dogs with the same conditioning and racing history were also examined after completing a multiple-day exercise session of 400 miles in 4 days, followed 6 hr later by a treadmill standardized exercise test.

Muscle biopsies were obtained from all dogs and prepared for the isolation of sarcolemmal vesicles. General anesthesia was induced and maintained using intravenous propofol; dogs completing a treadmill standardized exercise test were anesthetized within 2 min of completion of the exercise. Approximately 1 g of muscle was obtained through aseptic excisional biopsy from the gracilis muscle. Giant sarcolemmal vesicles were prepared as described by Luiken *et al.* [6]. Muscle was cut into thin strips and incubated for 1 hr at 34°C with shaking (100 rpm) in a solution of 140 mM KCl/10 mM MOPS (pH 7.4) with collagenase VII (150 units/ml) and aprotinin (10 mg/ml). The muscle was washed with additional volumes of KCl/MOPS containing 10 mM EDTA and the supernatants collected. Percoll, KCl and aprotinin were added to the supernatants to a final concentration of 3.5%, 28 mM and 10 µg/ml, respectively. The supernatant was placed as the bottom layer of a density gradient under 3 mL of 4% Nycodenz (middle layer) and 1 mL KCl/MOPS (upper layer), and centrifuged at 60 xg for 45 min at 25°C. The vesicles were harvested from the interface of Nycodenz and KCl/MOPS, washed with KCl/MOPS, and pelleted by centrifugation at 9000 xg for 10 min. The resulting pellet was resuspended in KCl/MOPS to a final protein concentration of 1.25 µg/ml.

Palmitate and glucose uptake were measured simultaneously according to previously-published techniques [6] by adding 40 µl of a reaction solution containing 14 µM palmitate (including 0.3 µCi of ^14^C-palmitate, Sigma Aldrich NEC534050UC, specific activity 850 mCi/mmol) and 5 mM glucose (including 0.3 µCi of ^3^H-3-O-methylglucose, Sigma Aldrich NET379001MC, specific activity: 80.2 Ci/mmol), and 0.1% bovine serum albumin in KCl/MOPS to 40 µL of vesicle solution containing 50 µg of protein (final transport volume 80 µL). Substrate uptake was terminated after 15 s by adding 1.4 ml of ice-cold KCl/MOPS containing 2.5 mM HgCl and 0.1% bovine serum albumin. The reaction tubes were centrifuged in a microfuge at maximum speed for 1 min, and the supernatant removed. The radioactivity in the tip of the tube was measured to determine vesicle uptake. A subset of vesicles that were mixed with the radio-substrate solution were disrupted using 3X freeze/thaw cycles prior to centrifugation to quantify the amount of non-specific substrate binding (i.e., binding to the vesicles without transport).

Whole muscle (50–60 mg) was homogenized using a Polytron in ice cold buffer (1 mL) containing Tris (50 mM), Triton X-100 (1% v/v), EGTA (1 mM), EDTA (1 mM), NaF (50 mM), sodium β-glycerol phosphate (10 mM), sodium pyrophosphate (5 mM), DTT (2 mM), sodium orthovanadate (1 mM), phenylmethylsulfonyl fluoride (1 mM), and aprotinin, leupeptin, and pepstatin A (10 µg/mL each). After homogenization, the solution was sonicated (15 s) and rocked for 30 min at 4°C. The solution was centrifuged at 1,500 xg for 15 min at 4°C and the supernatant collected. Protein concentration was determined by the Bradford method, the sample was combined with Lamelli buffer and 20 µg of protein was loaded for SDS-PAGE.

The protein concentration of the giant sarcolemmal vesicle samples was determined by the BCA method, the sample combined with Lamelli buffer and 10 µg of protein was loaded for SDS-PAGE. Proteins were separated by electrophoresis on a 7.5% SDS-polyacrylamide gel and transferred to a polyvinylidene difluoride membrane. Membranes were blocked for 1 h at room temperature (RT) in TBS-T containing 7.5% bovine serum albumin (BSA). Membranes were incubated overnight at 4°C in the appropriate primary antibody in TBS-T containing 7.5% BSA. Antibodies for GLUT4 was from Chemicon (Temecula, CA). Antibodies for FAT/CD36 (MO25) were gifts from N.N. Tandon. Membranes were incubated for 1 h (RT) with the corresponding secondary antibody, and the immune complex detected with enhanced chemiluminescence (PerkinElmer Life Science, Boston, MA) and quantified by densitometry (ChemiGenius2 Bioimaging, SynGene, Cambridge, UK). An internal standard was included on all blots and the relative densitometry values for the samples were expressed as a percentage of the standards on the corresponding gels. All blots were stained with Ponceau S (Sigma, Missiauga, ON) to ensure equal loading of protein.

The effect of conditioning on basal (unstimulated) transport or transporter expression was evaluated by comparing biopsy data obtained from rested unconditioned and rested conditioned dogs. The effect of conditioning on exercise-stimulated transport or transporter expression was determined by comparing data from post-exercise biopsies from unconditioned and conditioned dogs. The effect of treadmill exercise was determined by comparing basal and post-exercise biopsies in dogs with similar conditioning states. Finally, the effect of sustained multiday exercise on exercise-stimulated transport or transporter expression was evaluated by comparing biopsies from post-exercise conditioned dogs and post-exercise multiday exercise dogs. All comparisons were performed using a Student's t-test and p<0.05 was considered significant.

## Results

Western blot assays for abundance of sarcolemmal transporters in whole muscle were performed on tissues from 5 rested unconditioned dogs, 8 post-exercise unconditioned dogs, 8 rested conditioned dogs, 8 post-exercise conditioned dogs, and 7 post-exercise conditioned dogs following the 4 day exercise session. Insufficient tissue was obtained to prepare sarcolemmal vesicles in some instances, and therefore transport data were only obtained in some of the subjects. Sarcolemmal transport assays were performed on 4 rested unconditioned dogs, 3 post-exercise unconditioned dogs, 3 rested conditioned dogs, 4 post-exercise conditioned dogs, and 6 post-exercise conditioned dogs following the 4 day exercise session. Western blot assays for abundance of sarcolemmal transporters on the sarcolemma (using the sarcolemmal vesicles) were performed on samples from 6 rested unconditioned dogs, 8 post-exercise unconditioned dogs, 7 rested conditioned dogs, 8 post-exercise conditioned dogs, and 7 post-exercise conditioned dogs following the 4 day exercise session.

**Figure 1:**
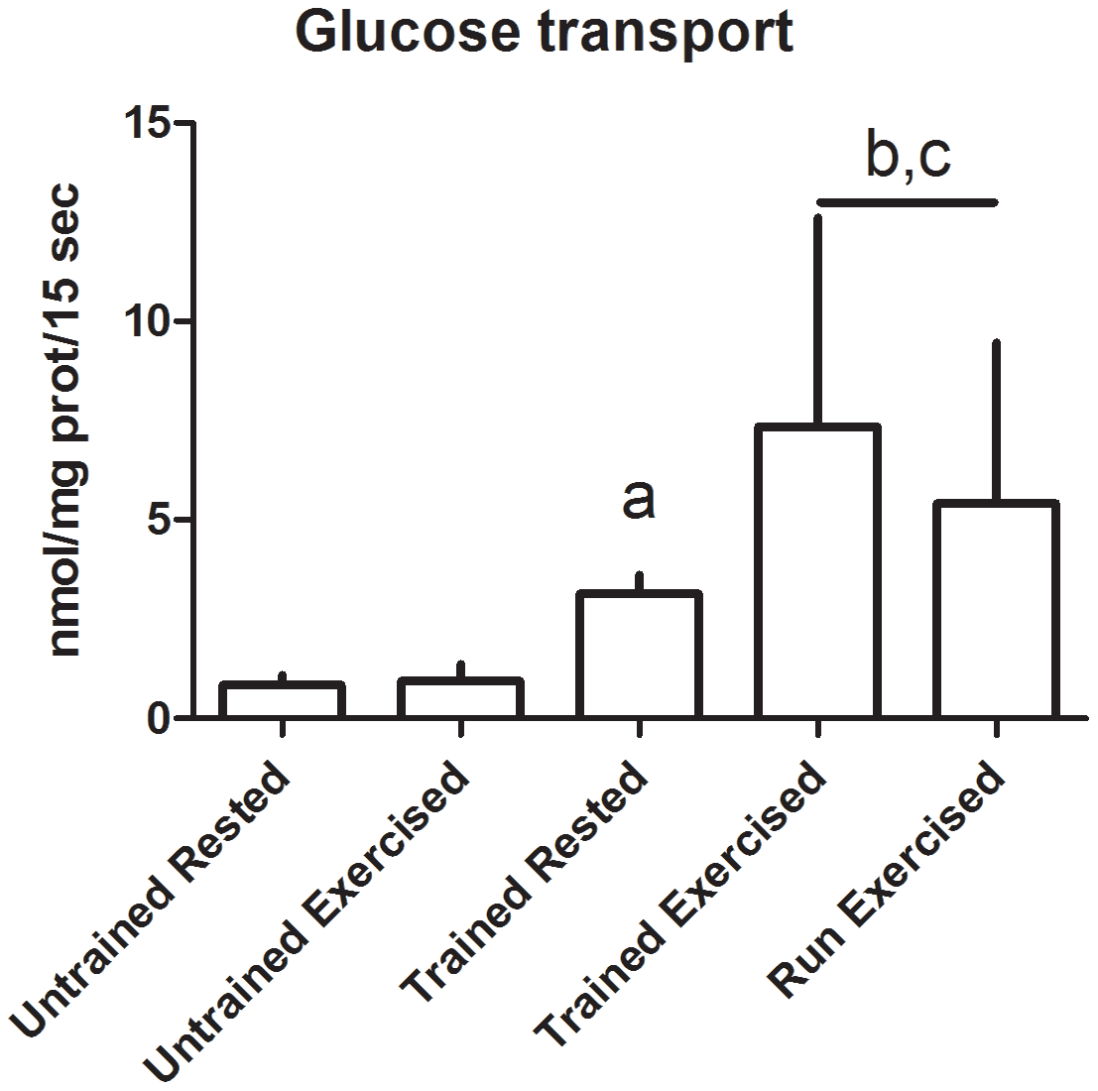
Transport rate of glucose across sarcolemmal vesicles. Data displayed as mean and standard deviation. Untrained Rested n = 4, Untrained Exercised n = 3, Trained Rested n = 3, Trained Exercised n = 4, Run Exercised n = 6. a: significantly different from Untrained Rested, p = 0.0029; b: significantly different from Untrained Exercised, p = 0.035; c: significantly different from Trained Rested, p = 0.029.

**Figure 2:**
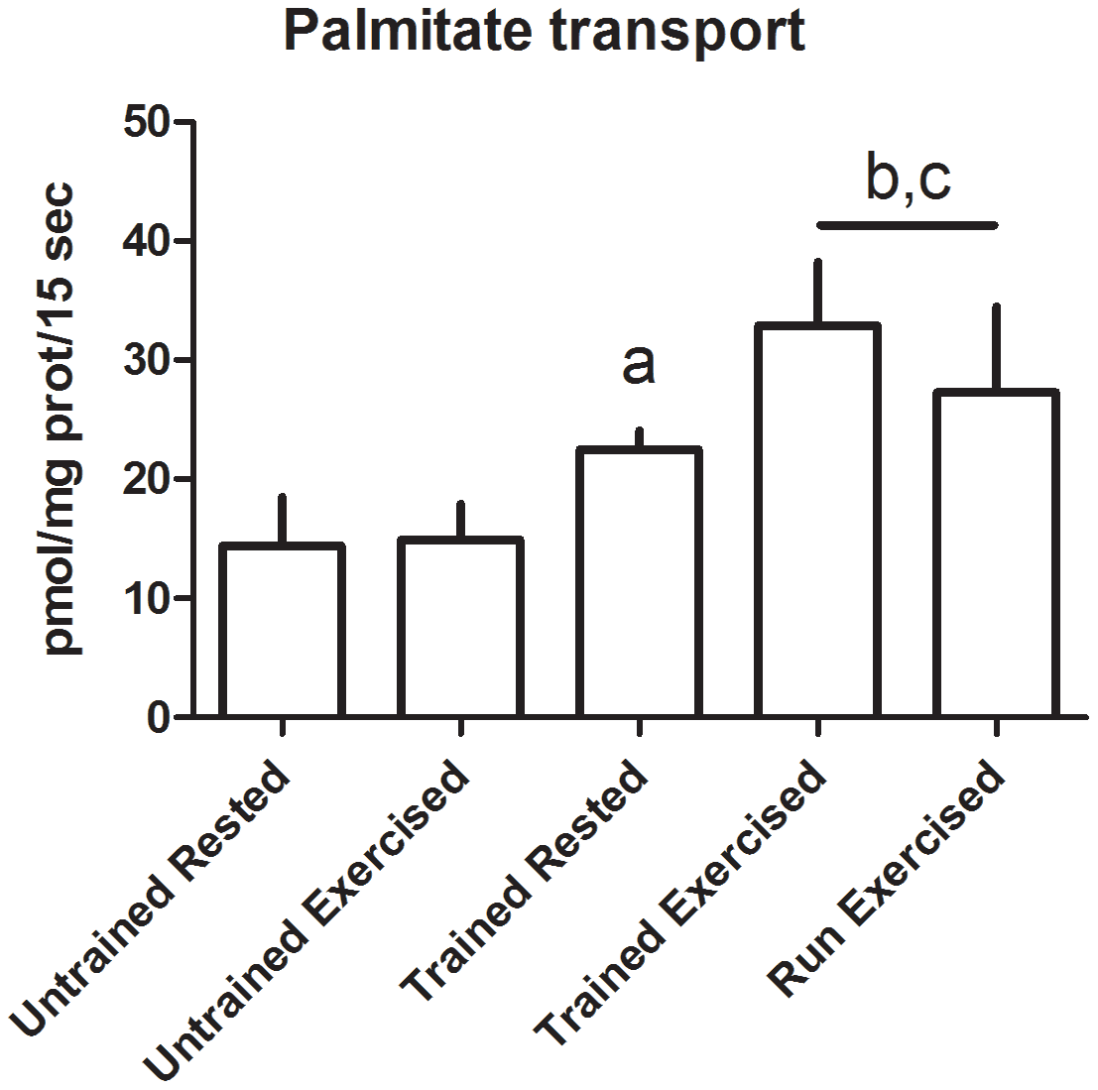
Transport rate of palmitate across sarcolemmal vesicles. Data displayed as mean and standard deviation. Untrained Rested n = 3, Untrained Exercised n = 4, Trained Rested n = 2, Trained Exercised n = 4, Run Exercised n = 6. a: significantly different from Untrained Rested, p = 0.031; b: significantly different from Untrained Exercised, p = 0.0015; c: significantly different from Trained Rested, p = 0.0091.

Multiday exercise did not result in a change in contraction-induced sarcolemmal transport of either glucose or palmitate when compared to similarly-conditioned dogs that were rested prior to treadmill exercise (Glucose: 7.35 (SD 5.26) vs 5.41 (SD 4.05) µmol/mg protein/15 sec, p = 0.28; Palmitate: 32.91 (SD 5.31) vs 27.27 (SD 7.20) pmol/mg prot/15 sec, p = 0.10) (Figures 1-2); therefore the data from these groups were combined for further evaluation of the effects of conditioning on sarcolemmal substrate transport.

Conditioning resulted in increased basal and contraction-stimulated glucose and palmitate transport. Basal transport of glucose increased 270% (0.84 (SD 0.25) vs 3.15 (SD 0.48) nmol/mg protein/15 sec, p<0.01) (Figure 1) and basal transport of palmitate increased by 60% (14.42 (SD 4.07) vs 22.48 (SD 1.59) pmol/mg prot/15 sec, p = 0.03) (Figure 2). Contraction stimulated transport of glucose increased 550% (0.95 (SD 0.43) vs 6.19 (SD 4.40) nmol/mg protein/15 sec, p = 0.04) (Figure 1) and contraction-stimulated transport of palmitate increased almost 100% (14.90 (SD 3.01) vs 29.52 (SD 6.83) pmol/mg prot/15 sec, p<0.01) (Figure 2). The standardized exercise test resulted in signficant increases in glucose and palmitate uptake in the conditioned dogs (p = 0.03 and <0.01, respectively), but not in the unconditioned dogs (p = 0.36 and 0.42, respectively).

**Figure 3:**
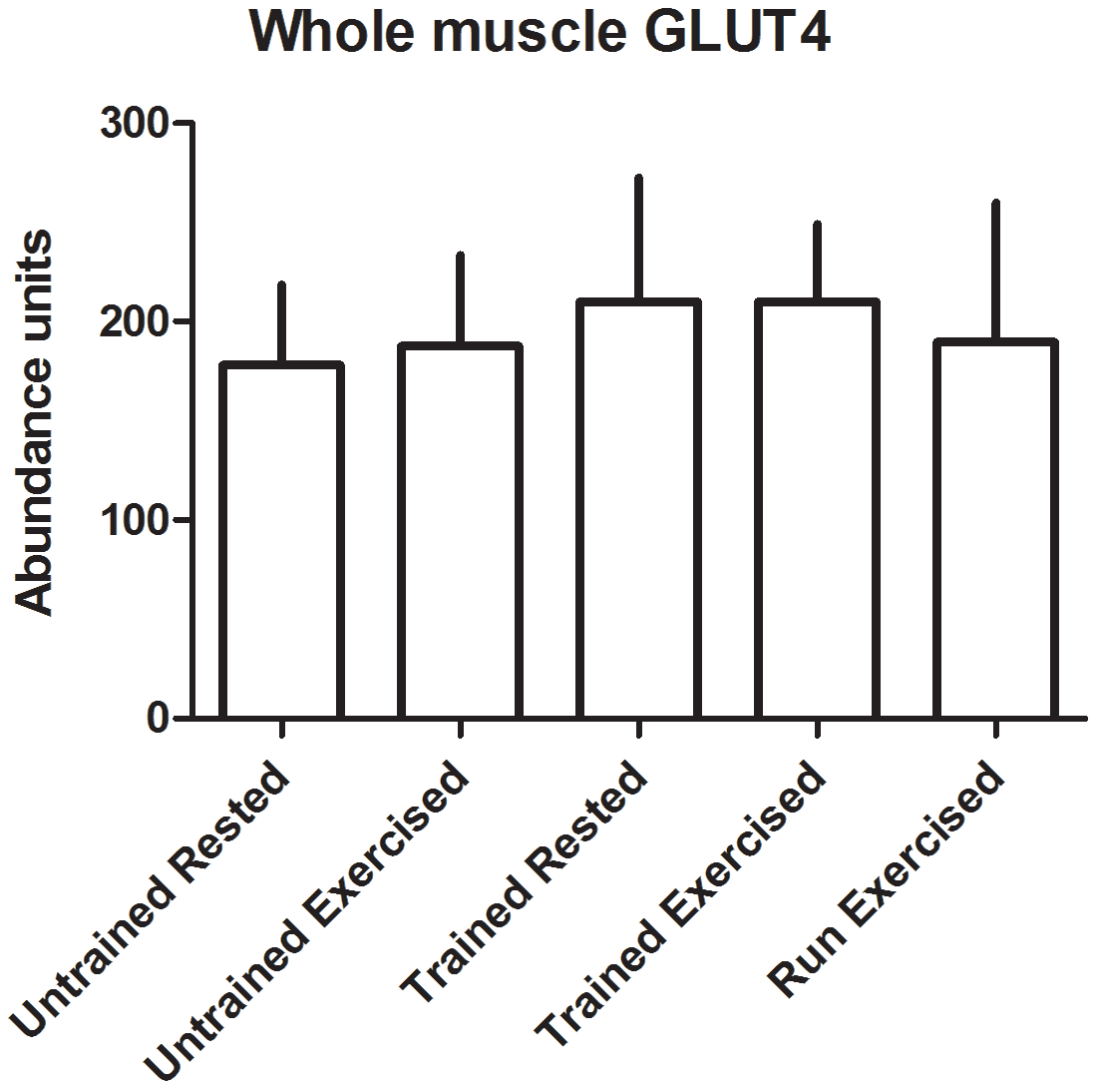
GLUT4 abundance in whole muscle homogenates. Transporter was quantified using Western blot, and abundance is expressed as arbitrary optical density units. Data displayed as mean and standard deviation. Untrained Rested n = 5, Untrained Exercised n = 8, Trained Rested n = 8, Trained Exercised n = 8, Run Exercised n = 7.

**Figure 4:**
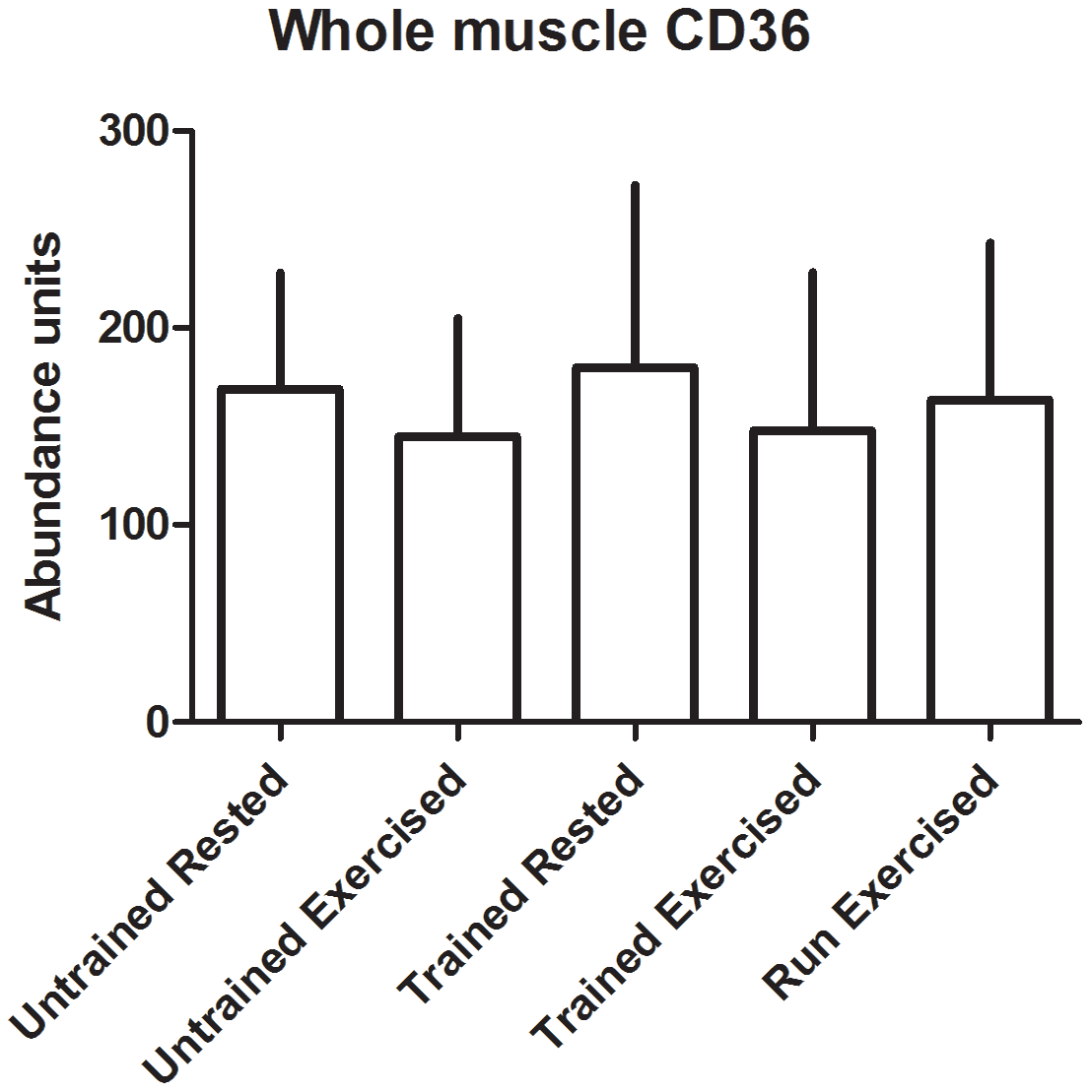
FAT/CD36 abundance in whole muscle homogenates. Transporter was quantified using Western blot, and abundance is expressed as arbitrary optical density units. Data displayed as mean and standard deviation. Untrained Rested n = 5, Untrained Exercised n = 8, Trained Rested n = 8, Trained Exercised n = 8, Run Exercised n = 7.

**Figure 5:**
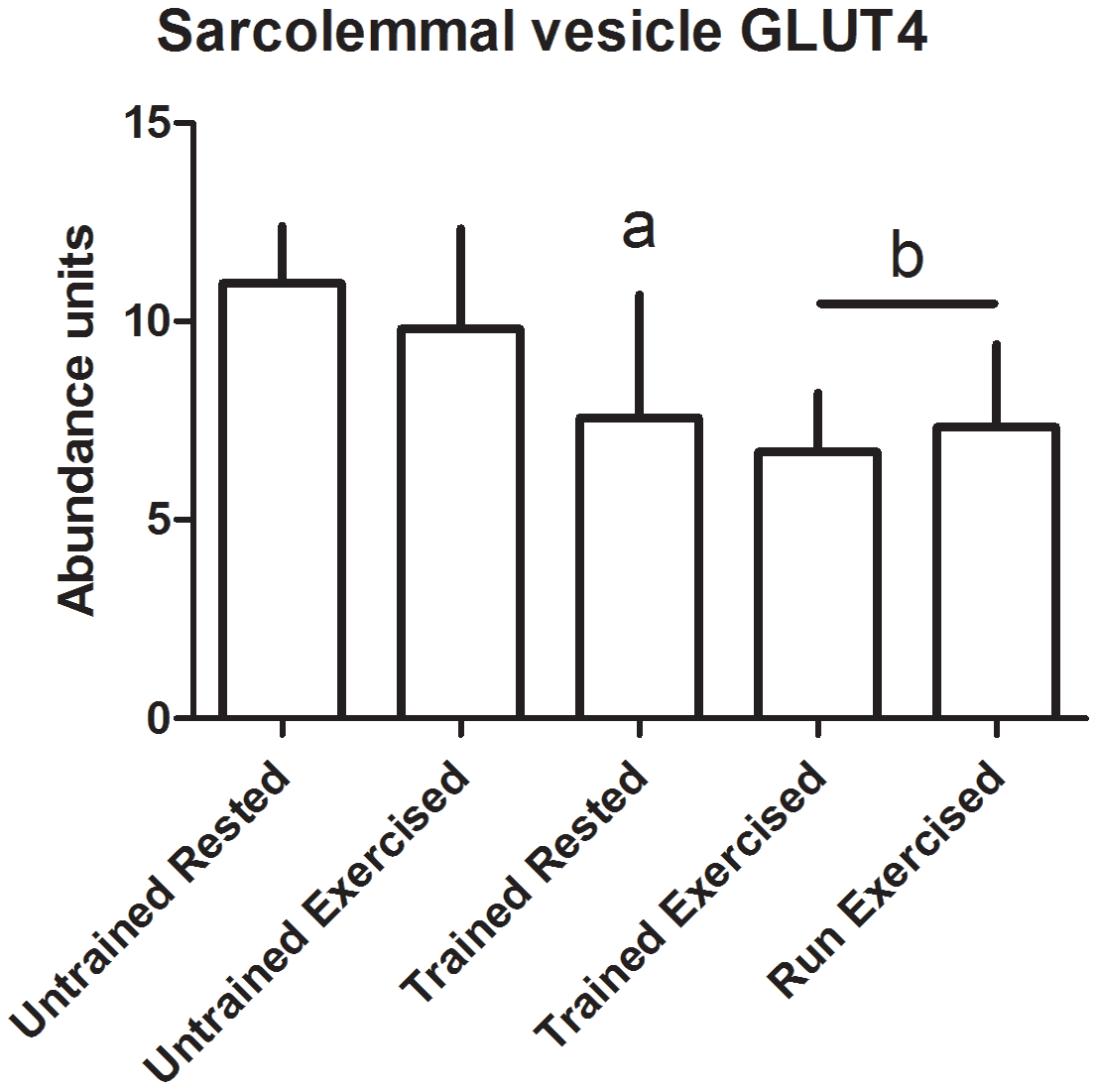
Plasma membraneGLUT4 abundance. Transporter was quantified using Western blot, and abundance is expressed as arbitrary optical density units. Data displayed as mean and standard deviation. Untrained Rested n = 6, Untrained Exercised n = 8, Trained Rested n = 7, Trained Exercised n = 8, Run Exercised n = 7. a: significantly different from Untrained Rested, p = 0.015; b: significantly different from Untrained Exercised, p = 0.0025.

**Figure 6:**
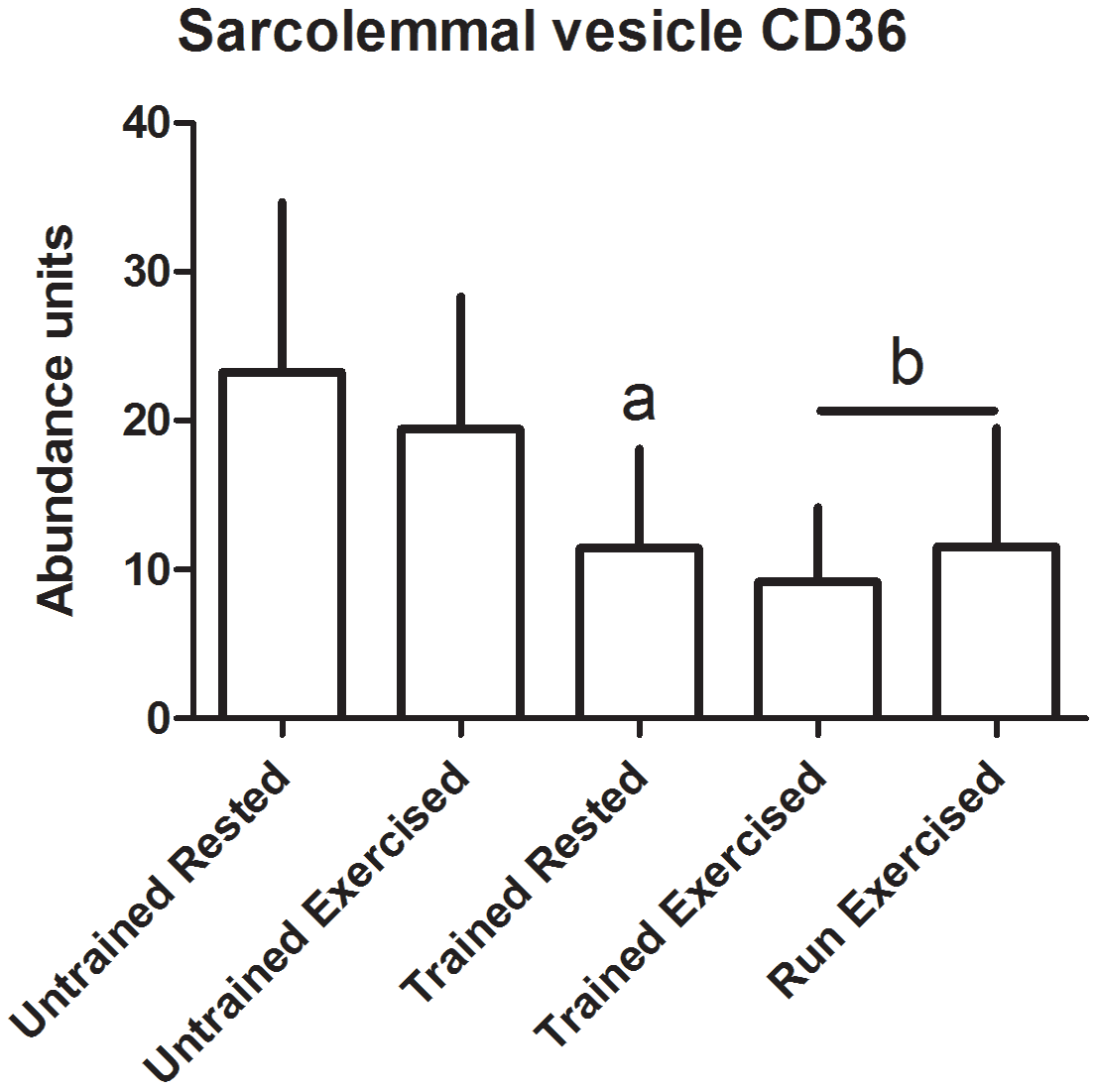
Plasma membraneFAT/CD36 abundance. Transporter was quantified using Western blot, and abundance is expressed as arbitrary optical density units. Data displayed as mean and standard deviation. Untrained Rested n = 6, Untrained Exercised n = 8, Trained Rested n = 6, Trained Exercised n = 8, Run Exercised n = 7. a: significantly different from Untrained Rested, p = 0.030; b: significantly different from Untrained Exercised, p = 0.0048.

There were no significant changes in transporter abundance in the whole muscle homogenates from any of the groups (Figures 3-4). In the sarcolemmal vesicles, conditioning resulted in decreased presence of GLUT4 and FAT/CD36 at the plasma membrane in rested dogs (p = 0.02 and 0.03, respectively) and in post-exercise dogs (p<0.01 in both) (Figures 5-6). There was no effect of multiday exercise on contraction-mediated sarcolemmal levels of either transporter (p = 0.26 for both), and there was no significant effect of contraction on sarcolemmal levels of either transporter within a conditioning state (GLUT4 unconditioned p = 0.15, conditioned p = 0.33; FAT/CD36 unconditioned p = 0.26, conditioned p = 0.36) (Figures 5-6).

## Discussion

The physiological limitations to exercise performance can be different for different types of exercise. In the case of prolonged submaximal exercise, the availability of macromolecular oxidizable substrates such as glucose and fatty acid can be limiting. Intramuscular stores of these substrates are finite, and if a given exercise intensity results in the reliance on these stores, then that exercise intensity can not be performed for prolonged periods. Conversely, in order to perform such exercise for prolonged periods, an alternate strategy for the provision of oxidizable substrates is necessary. Our previous studies of racing Alaskan sled dogs have suggested that dogs conditioned for ultra-endurance competition – races that span multiple days – develop just such an alternative strategy when performing exercise that would otherwise rely on finite stores of intramuscular substrate. Our primary hypothesis was simple – that the dogs rapidly increase their capacity for sarcolemmal transport of blood-borne substrates. The results of our studies partially support our original hypothesis, but also provide some unusual findings that raise questions regarding the metabolic strategy of a racing sled dog to meet its energetic demands.

The specific phenomenon that we sought to explain with this series of experiments was the rapid conversion from a glycogen-depleting to a glycogen-sparing phenotype described in our previous studies [4], [5]. In our previous studies, the exercise challenges were conducted after approximately 4 months of conditioning but no multiday race, compared to the current study in which the dogs had completed 7 months of conditioning and multiple races, ranging from 300 miles to 1000 miles. It is possible, perhaps even likely, that the metabolic strategy present in the conditioned dogs of the current study was similar to the exercise-induced glycogen-sparing phenotype present at the end of the previous studies and as a result, the additional 400-mile exercise challenge imposed in this study was not sufficiently stressful, from a metabolic perspective, to induce any further adaptation. This aspect of the experimental design was unavoidable due to the greater invasiveness of the biopsy technique required for sarcolemmal vesicle preparation, but serves as a caution that the magnitude of conditioning may not be comparable between the current study and previous studies of endurance sled dogs.

A season of aerobic conditioning resulted in a significant increase in the resting (basal) sarcolemmal transport of both glucose and palmitate. The increased basal transport of glucose and palmitate are unlikely to be due to the effects of post-prandial insulin since at the time of examination, the dogs had not been fed for at least 12 h. It is also unlikely to be due to residual effects of acute exercise as the dogs had been rested for at least 96 h and previous studies have shown that 96 h following a prolonged, glycogen depleting exercise bout, muscle glycogen concentrations have returned to baseline values [5]. It is possible that the increase in basal transport measured in this study is not present *in vivo*. Although expression of sarcolemmal GLUT4 is considered the rate-limiting step for muscle glucose uptake at rest, phosphorylation of intracellular glucose by hexokinase II is believed to be the rate-limiting step during exercise [1]. It is possible that in highly-conditioned dogs, the latter regulatory approach predominates even at rest. In this scenario, sarcolemmal transport of glucose is limited by down-regulation or inhibition of hexokinase II, resulting in increased intracellular concentrations of glucose and loss of the trans-sarcolemmal concentration gradient. Sarcolemmal flux of glucose would thus be reduced despite increased expression of transporters on the sarcolemma. Alternatively, the measured increase in basal substrate uptake suggests an increased metabolic activity of the muscle. The nature of this increased activity, if it exists, is unknown. Lacking a recent history of strenuous exercise that might induce a conditioning response, we would not expect increased metabolic activity secondary to *de novo* protein synthesis. Rather, the most likely explanation would be increased energetic costs of maintenance of the conditioned phenotype, which also could also help explain why such a phenotype is not maintained when it is not needed during deconditioning. However, the magnitude of the substrate transport increases are substantial (3-fold increase in glucose transport and ∼50% increase in fatty acid transport), making it unlikely to be explained entirely by increased metabolic requirements of the conditioned muscle.

The effect of exercise on substrate transport was profoundly affected by conditioning. With the translocation of both GLUT4 and FAT/CD36 being contraction-mediated [7], [8], resulting in increased substrate transport [9], the lack of an exercise effect on substrate transport in unconditioned dogs was somewhat surprising. However, the intensity of our exercise test was relatively modest by the standards of even unconditioned sled dogs, whose initial conditioning activities may last 2-3 h at speeds 50% greater than our exercise test and while pulling a load. Exercise-induced translocation of GLUT4 increases as the duration of the exercise challenge increases [9], so the 20 min of exercise used in this study may not have been sufficiently strenuous to stimulate increased transarcolemmal movement of substrate. That the same modest intensity exercise resulted in large increases in substrate transport in conditioned dogs suggests that an increase in the gain of contraction-mediated substrate uptake is a major feature of endurance conditioning in dogs. Our finding of increased sarcolemmal transport of glucose during submaximal exercise in response to endurance conditioning is in contrast to a similar study in humans which found decreased sarcolemmal glucose transport [10]. Fatty acid transport was not measured in that study, but presumably the sarcolemmal transport of fatty acids increased to fuel the exercise challenge. It is possible that the increase in sarcolemmal transport capacity of glucose is misleading as the increased permeability of the sarcolemma to glucose may be offset by insufficient hexokinase II activity to maintain a large diffusion gradient for glucose [11]. However, a similar regulatory point for fatty acids has not been described and the increase in the gain of contraction-mediated fatty acid transport reported here is consistent with the general conclusion that endurance training increases fatty acid utilization at moderate workloads.

Endurance conditioning did not increase the abudance of either GLUT4 or FAT/CD36 in skeletal muscle. Previous studies in humans [12], [13], rats [14], and horses [15] have documented increased skeletal muscle content of GLUT4 following exercise conditioning, as well as increased FAT/CD36 in humans [16] and horses [17]. While surprising, these results are in agreement with our unpublished data showing a lack of upregulation of the corresponding genes during endurance conditioning in sled dogs. These data suggest that regulation of sarcolemmal substrate transport in dogs is accomplished more through alterations in the intrinsic activity of the components of substrate transport pathways, rather than synthesis and turnover of the components themselves.

Perhaps the most intriguing result from this study is the apparent lack of concordance between the substrate sarcolemmal transport data and the data relating to the sarcolemmal abundance of the putative transporters of these substrates. Numerous studies have reported concurrent increases in sarcolemmal expression of substrate-specific transporters and increased sarcolemmal transport of those substrates [1], [18], and this body of work is sufficiently robust to support the general conclusion that increases in sarcolemmal transport of specific substrates is the result of increased numbers of transporters expressed on the sarcolemma. The results presented herestand in stark contrast to this conclusion. In fact, to a large degree the changes transport data and transporter abudance data are opposite; the detected abundance of the transporters goes down as transport increases. Two possible explanations exist. First, that the transporters we selected to represent glucose and fatty acid transport (GLUT4 and FAT/CD36, respectively) are not the prevailing transporters for these substrates in these dogs and instead other, unmeasured transporters are responsible for the changes in substrate flux resulting from conditioning and exercise. Of all the identified fatty acid transport proteins, FAT/CD36 is the logical vehicle for dynamically-regulated fatty acid transport across the sarcolemma given its high capacity relative to the other fatty acid transport proteins [19] and the fact that its expression on the sarcolemma is upregulated by muscle contraction [7]. Similarly, GLUT4 is the most abundant glucose transporter identified in skeletal muscle and, like FAT/CD36, demonstrates increased sarcolemmal expression in response to muscle contraction [8]. Certainly, there are other possible transporters for both glucose and fatty acids, but unfortunately we lacked the sample volume to perform an exhaustive characterization of all possible transporters.

A second possibility is that the selected transporters undergo post-translational modification that increases the intrinsic activity of the transporters, thus requiring fewer transporters on the sarcolemmal to meet the metabolic demands of the contracting muscle. Phosphorylation of GLUT4 reduces the intrinsic activity of the transporter and one of the effects of insulin (in addition to stimulating transporter translocation to the cell membrane) is to dephosphorylate GLUT4 to increase intrinsic activity [20]. This effect can be inhibited by high intracellular calcium and by palmitate [21]. Reduced intrinsic activity of GLUT4 by high cytoplasmic ATP also has been suggested by molecular modelling [22]. There is also evidence to suggest that association of glyceraldehyde-3-phosphate dehydrogenase with the GLUT4 transporter increases the intrinsic activity of the latter [23], [24]. Less is known regarding the intrinsic activity of FAT/CD36, but there is some evidence that increased fatty acid transport activity of this transporter is related to its association with calveolae and lipids rafts [25]. Lack of concordance between substrate transport and transporter abundance in contracting muscle has been previously reported for lactate flux and monocarboxylate transporters [26], presumably due to a change in the intrinsic activity of these transporters, establishing at least the fact that such a change in the intrinsic activity of other transporters is a tenable possibility.

Our study demonstrates that endurance conditioning in racing sled dogs results in increased capacity for sarcolemmal transport of oxidizable substrates, but raises the possibility that in vivo, this transport capacity may be in excess of demand and actual transsarcolemmal flux of these substrates are regulated elsewhere in the transport pathway. Furthermore, our data suggests, albeit indirectly, that the increased transport capacity is due to modification of existing transporters instead of de novo synthesis of new transporters. Direct demonstration of this feature of exercise conditioning on substrate metabolism will be the focus of additional studies.
